# Evidence for Fast Electron Transfer between the High-Spin Haems in Cytochrome *bd*-I from *Escherichia coli*

**DOI:** 10.1371/journal.pone.0155186

**Published:** 2016-05-06

**Authors:** Sergey A. Siletsky, Fabrice Rappaport, Robert K. Poole, Vitaliy B. Borisov

**Affiliations:** 1 Belozersky Institute of Physico-Chemical Biology, Lomonosov Moscow State University, Moscow, Russian Federation; 2 Institut de Biologie Physico-Chimique, Unite Mixte de Recherche 7141 CNRS, Universite Paris 6, Paris, France; 3 Department of Molecular Biology and Biotechnology, The University of Sheffield, Sheffield, United Kingdom; University of Alberta, CANADA

## Abstract

Cytochrome *bd*-I is one of the three proton motive force-generating quinol oxidases in the O_2_-dependent respiratory chain of *Escherichia coli*. It contains one low-spin haem (*b*_558_) and the two high-spin haems (*b*_595_ and *d*) as the redox-active cofactors. In order to examine the flash-induced intraprotein reverse electron transfer (the so-called ''electron backflow''), CO was photolyzed from the ferrous haem *d* in one-electron reduced (*b*_558_^3+^*b*_595_^3+^*d*^2+^-CO) cytochrome *bd*-I, and the fully reduced (*b*_558_^2+^*b*_595_^2+^*d*^2+^-CO) oxidase as a control. In contrast to the fully reduced cytochrome *bd*-I, the transient spectrum of one-electron reduced oxidase at a delay time of 1.5 μs is clearly different from that at a delay time of 200 ns. The difference between the two spectra can be modeled as the electron transfer from haem *d* to haem *b*_595_ in 3–4% of the cytochrome *bd*-I population. Thus, the interhaem electron backflow reaction induced by photodissociation of CO from haem *d* in one-electron reduced cytochrome *bd*-I comprises two kinetically different phases: the previously unnoticed fast electron transfer from haem *d* to haem *b*_595_ within 0.2–1.5 μs and the slower well-defined electron equilibration with τ ~16 μs. The major new finding of this work is the lack of electron transfer at 200 ns.

## Introduction

Cytochrome *bd*-I is one of the three terminal oxidases in the aerobic electron transport chain of *Escherichia coli* [1–4]. The enzyme catalyzes the reduction of molecular oxygen to water with quinol [5]. The energy released in this redox reaction is stored in the form of a proton electrochemical gradient [6–12]. Apart from energy conservation, a *bd*-type oxidase serves other vitally important physiological functions [13, 14] including its contribution to bacterial resistance to nitric oxide, hydrogen peroxide, peroxynitrite [15–19], nitrite [20, 21], and sulfide [22]. Cytochrome *bd*-I contains three subunits, CydA (57 kDa), CydB (43 kDa) and CydX (4 kDa). A newly discovered small polypeptide CydX is thought to be required for maintenance of enzymatic activity and stabilization of the haems [23–27]. Cytochrome *bd*-I carries one low-spin haem (*b*_558_) and the two high-spin haems (*b*_595_ and *d*). In the course of a catalytic reaction, an electron from quinol transfers to haem *b*_558_ and then to the oxygen reductase active site. The organization of the oxygen reductase site remains unclear. It certainly comprises haem *d* at which O_2_ is bound, activated and reduced to 2H_2_O. Whether the site also contains the second haem, *b*_595_, is not known with certainty. A large body of spectroscopic data suggests that haem *b*_595_ indeed can form with haem *d* a di-haem active site [8, 10, 28–39] and that cytochromes *b*_595_ and *b*_558_ are each oxidised as the ‘oxy’ form decays (650 nm) during the low-temperature reaction with oxygen [40]. However, according to other reports, haem *b*_595_ has an alternative or additional function [2, 41–43]. As to a possible additional role for haem *b*_595_, it's also worth mentioning that the observed catalase activity of cytochrome *bd*-I from *E*. *coli* could be associated with this haem [44]. Thus the exact functions of the high-spin haem *b*_595_ remain to be clarified. To better understand the role of haem *b*_595_ in the intraprotein electron transfer and the oxygen reduction reaction, we have applied time-resolved transient absorption spectroscopy and spectral modeling to the interhaem electron backflow reaction in cytochrome *bd*-I.

Flash photolysis of CO from the “mixed-valence” form of a terminal oxidase is a method by which haem-to-haem electron transfer can be measured. In cytochrome oxidase, initially the haem *a*_3_/Cu_B_ binuclear site is trapped in its reduced ferrous/cuprous state and stabilized by CO binding to the haem iron whereas the two other redox sites, haem *a* and Cu_A_, are oxidized. Flash photolysis of CO results in ultrafast displacement of CO from haem *a*_3_ (within a fraction of a picosecond) to bind Cu_B_ which in turn gives rise to the three phases of the reverse electron transport (the so-called ''electron backflow'') at neutral pH. The electron re-equilibration should occur first by the electron transfer to haem *a* (in two steps with *τ* ~1.4 ns and ~3 *μ*s) and then from both haems to Cu_A_ (~35 *μ*s). The 3-*μ*s phase was reported to be rate-limited by CO dissociation from Cu_B_ [45], whereas the 1.4-ns and 35-*μ*s phases are rate-limited by the electron tunneling between haem *a*_3_ and haem *a* and between haem *a* and Cu_A_, respectively [46–48]. The nanosecond interhaem electron transfer was detected in both cytochrome *c* and quinol oxidases of the haem-copper superfamily [46, 49, 50]. Under similar conditions, the 16-μs component of the photolysis-induced electron backflow was resolved in cytochrome *bd*-I [39]. In this work, we provide, to the best of our knowledge for the first time, evidence for the existence of a faster electron backflow component, on a submicrosecond time range, induced by photodissociation of CO from ferrous haem *d* in one-electron-reduced state of cytochrome *bd*-I.

## Materials and Methods

### Biological materials

Membrane vesicles were prepared by passing the *E*. *coli* cells (strain GO105/pTK1) through a French press according to [51]. Cytochrome *bd*-I was isolated and purified as reported in [51, 52].

### Sample preparation

One-electron-reduced CO-bound cytochrome *bd*-I (*b*_558_^3+^*b*_595_^3+^*d*^2+^-CO) was prepared as described in [33, 37, 39]. To do this, the as-prepared cytochrome *bd*-I that is mostly in the one-electron-reduced O_2_-bound state i.e. *b*_558_^3+^*b*_595_^3+^*d*^2+^-O_2_, was first purged with argon and subsequently equilibrated with 1 atm CO. To obtain the fully reduced CO-bound cytochrome *bd*-I (*b*_558_^2+^*b*_595_^2+^*d*^2+^-CO), the oxidase was pre-reduced for 30 min with a few grains of solid sodium dithionite and then equilibrated with 1 atm CO. The sample subjected to photolysis contained the CO (1 mM) complex with cytochrome *bd*-I (1.85 μM) in 50 mM Hepes, 50 mM Ches, 0.1 mM EDTA, 0.05% sodium *N*-lauryl-sarcosinate, pH 8.0, 20°C.

### Cytochrome *bd*-I concentration

Oxidase concentration was determined from the difference absorbance spectrum (dithionite-reduced *minus* “air-oxidized”) using Δ*ε*_628–607_ of 10.8 mM^−1^ cm^−1^ [32].

### Spectroscopy

For CO photolysis, nanosecond pulses were used with time duration 5–15 ns and excitation wavelengths at 532 nm (from a Nd YAG laser) and 640 nm (from a Nd YAG pumped dye laser), the latter wavelength being near the haem *d* α-band maximum. The photoinduced absorption changes were measured with a single beam home-built spectrophotometer with submicrosecond time resolution (for details see [39, 53–55]) and with a nanosecond spectrophotometer, as described in [37, 56].

### Data analysis

Origin (OriginLab Corporation) and MATLAB (The Mathworks, South Natick, MA) were used for data manipulation and presentation.

## Results and Discussion

**Fig 1:**
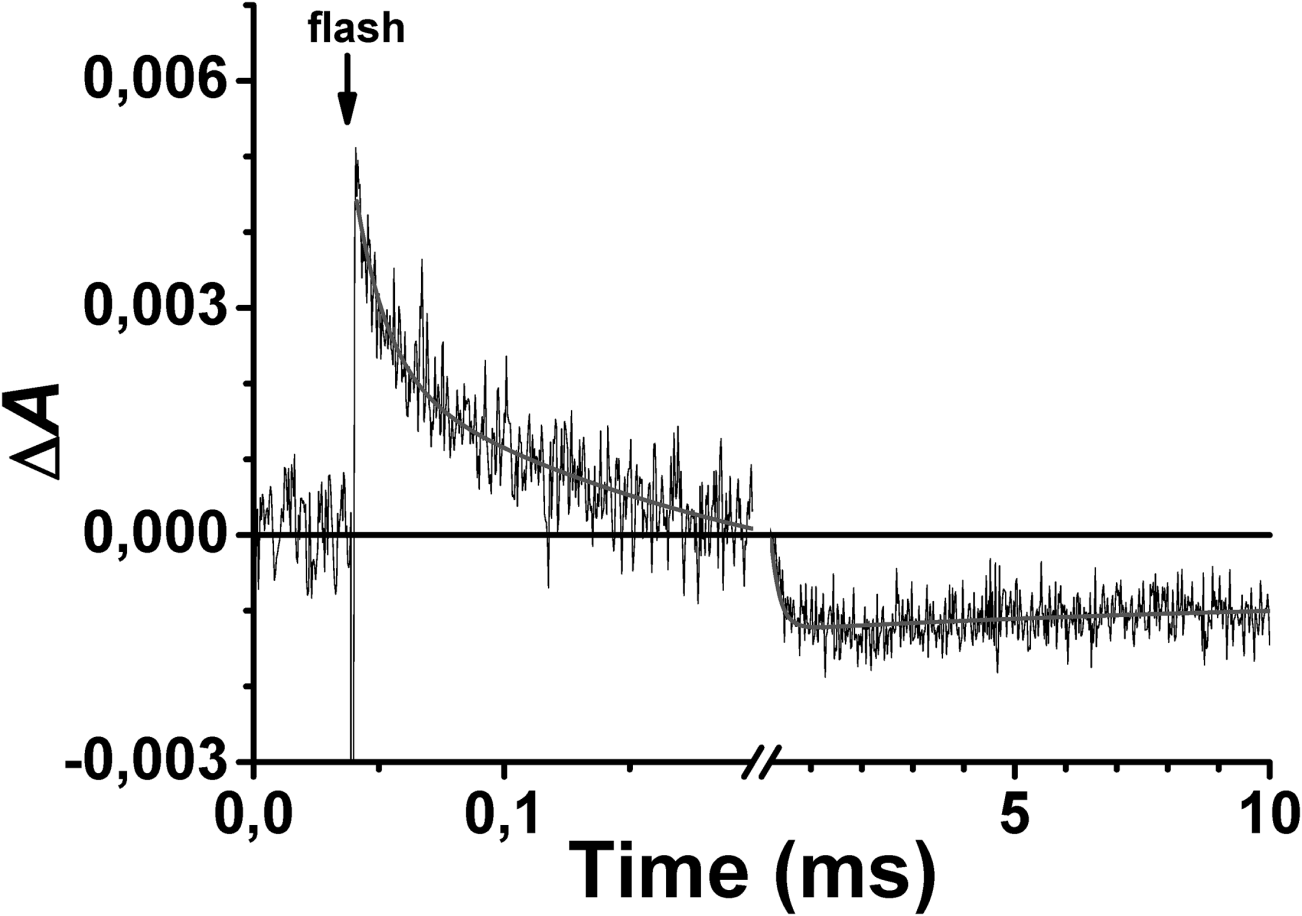
The 432 nm absorption change associated with flash-induced dissociation and subsequent recombination of CO with one-electron-reduced cytochrome *bd*-I from *E*. *coli*. The data points (noisy trace) are shown with their best fit (smooth line).

Laser flash-photolysis of one-electron-reduced CO-poised cytochrome *bd*-I causes immediate dissociation of CO from ferrous haem *d*. CO photolysis is followed by its recombination with cytochrome *bd*-I and the latter process can be fitted by three exponentials with apparent first-order rate constants (*k*) of 6.5×10^4^ s^-1^, 5.5×10^3^ s^-1^ and 3.3 ×10^1^ s^-1^. The kinetic trace at 432 nm at which all the transitions can be seen is depicted in Fig 1. Previous work [39] allowed one to compose and assign the spectra of these kinetic steps. The rapid phase (*k* = 6.5×10^4^ s^-1^ at 1 mM CO) is assigned to bimolecular recombination of CO to haem *d* plus backflow of the electron from haem *d* to haem(s) *b*. The intermediate phase (*k* = 5.5×10^3^ s^-1^) is due to return of the electron from haems *b* to haem *d* and bimolecular recombination of CO in that enzyme fraction. The slow phase (*k* = 3.3 ×10^1^ s^-1^) is complex but dissociation of an unidentified ligand (L) from haem *d* is possibly a major contributing factor [39].

**Fig 2:**
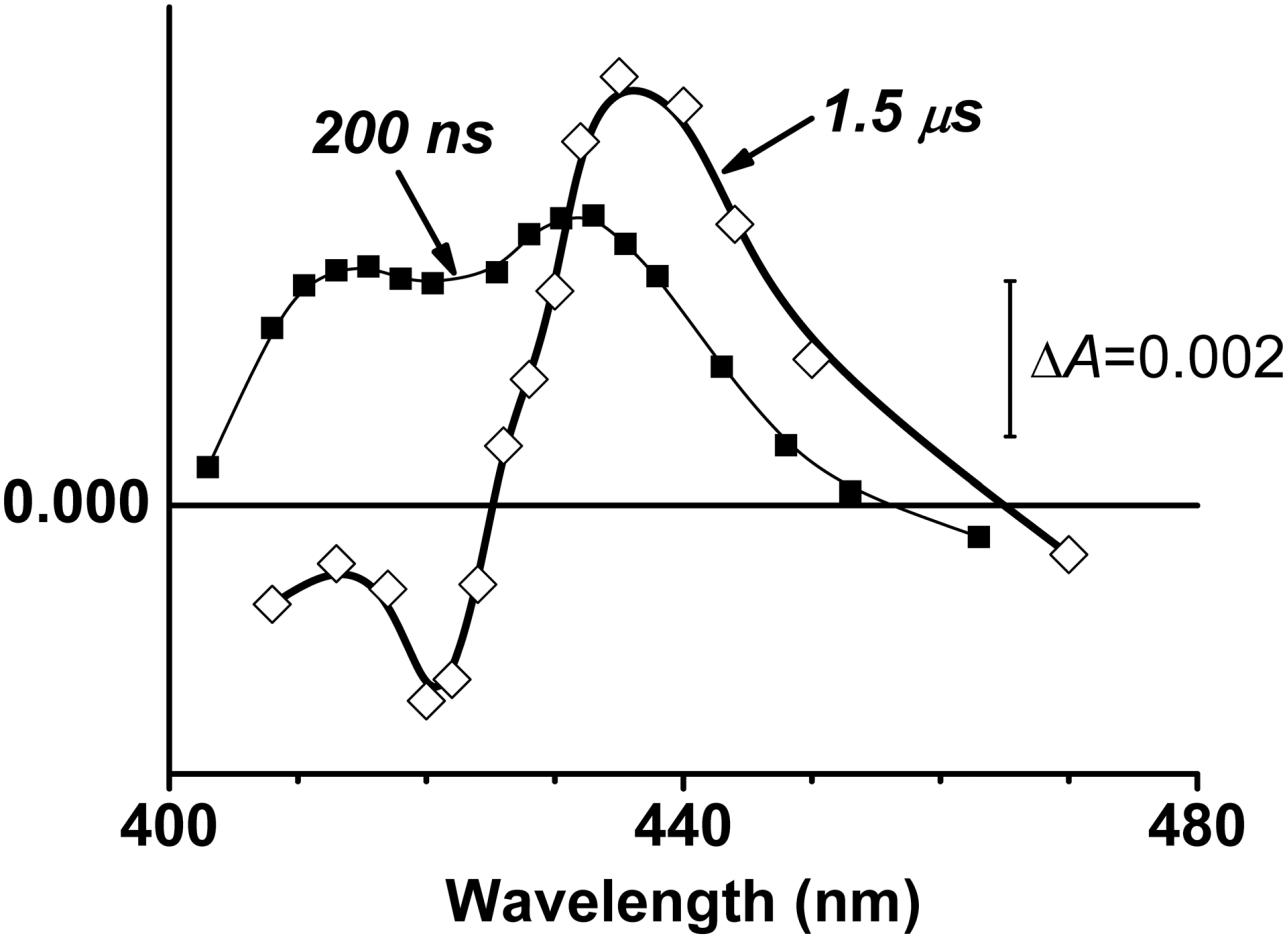
Transient difference absorption spectra of one-electron-reduced cytochrome *bd*-I after photolysis of CO at delay times of 200 ns and 1.5 μs.

Fig 2 shows clearly distinct transient absorption spectra at delay times of 1.5 μs (thick line plus open diamond symbols) and 200 ns (thin line plus filled square symbols). The 1.5-μs spectrum has two pronounced extrema, a maximum at about 437 nm and a minimum at 420 nm. This spectrum reflects the almost pure electron transfer reaction. As suggested by a previous report [39], in this redox state of the enzyme, dissociation of CO from the distal side of haem *d* may be accompanied by simultaneous binding of a ligand of unknown structure (L) to the proximal side of haem *d*. Thus, the absorption changes caused by these two processes mostly cancel each other out. L is then dissociated from haem *d* in the slow phase to return the haem ligation state to that before photolysis.

The identity of L is to be determined. To date, we favour a hypothesis that L is an internal protein ligand. Under this assumption, the easiest option could be that L is the proximal ligand to haem *d*. The haem *d* axial ligand is not yet identified. We assume that it should be a weak ligand like a glutamate residue [57] that could be easily detached from the haem iron rather than a strongly coordinated histidine residue [58]. This proposal is consistent with an earlier suggestion that the native ligand of haem *d* is replaced with cyanide to produce the five-coordinate high-spin cyano adduct [59, 60]. The fact that absorption changes that possibly reflect exchange or binding of an internal ligand to haem *d* in the course of reduction of the *bd* enzyme are similar to the changes caused by CO [61] is also in line with this proposal.

The 1.5-μs transient spectrum evidently is not identical to that at a delay time of 200 ns after CO photolysis from the same initial, one-electron-reduced state of cytochrome *bd*-I (Fig 2). The 200-ns spectrum displays a double-humped band with the two local maxima, at 415 and 432 nm, but has no clear minimum. Obviously, some event characterized by the change in absorption of the haems has occurred between 200 ns and 1.5 μs.

**Fig 3:**
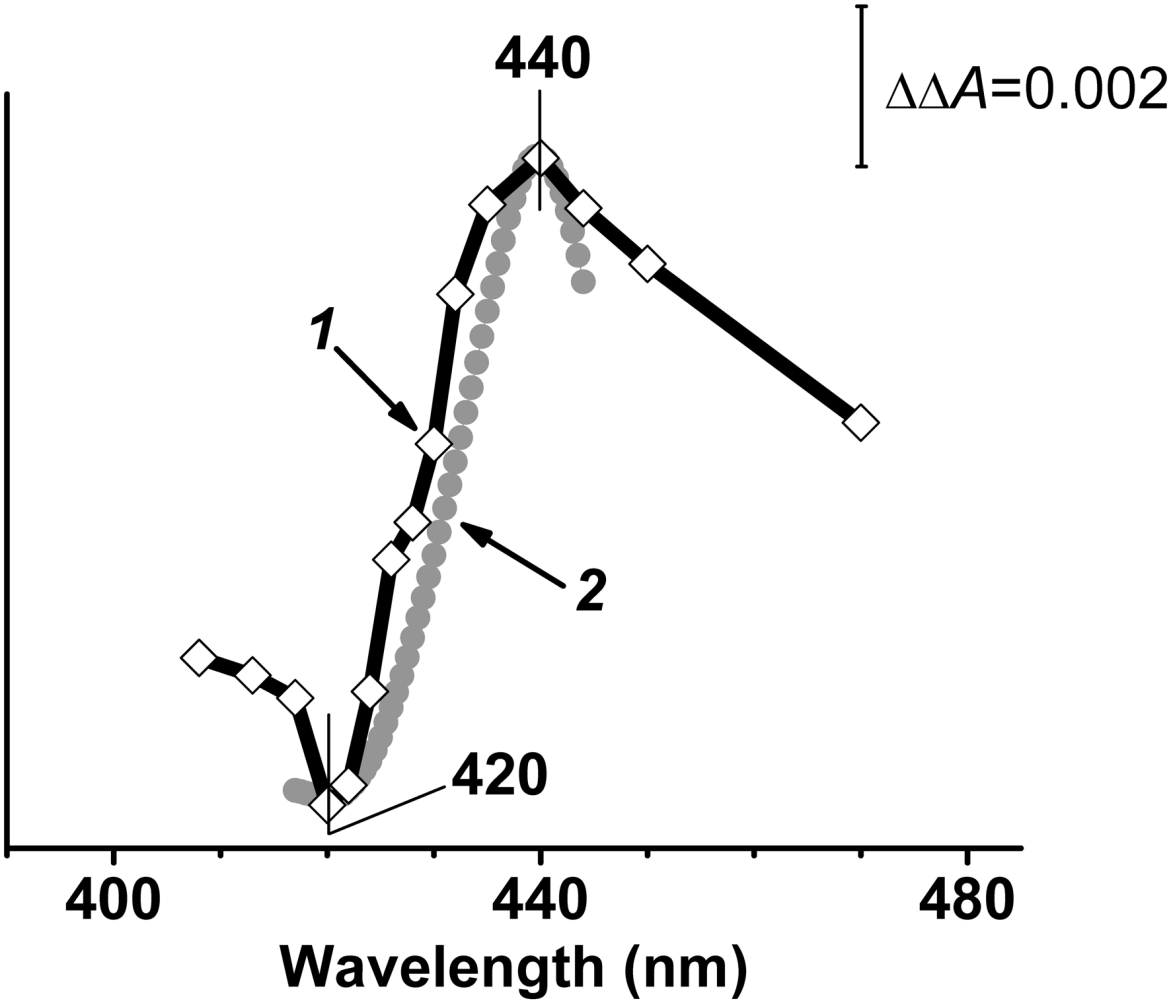
Simulation of the spectrum of electron backflow in one-electron-reduced cytochrome *bd*-I. Shown are double difference spectra: (*1*) Difference between transient spectra at delay times of 1.5 μs and 200 ns shown in Fig 2. (*2*) Model spectrum of electron transfer from haem *d* to haem *b*_595_ computed using reduced-*minus*-oxidized difference absorption spectra of haem *d* and haem *b*_595_ reported in [62].

Fig 3 shows a difference between the 1.5-μs and 200-ns transient absorption spectra (line *1*). The difference can be reasonably fitted by the spectrum of electron transfer from haem *d* to haem *b*_595_ (Fig 3, line *2*). Thus, photodissociation of CO from haem *d* is followed by fast (between 0.2 and 1.5 μs) electron backflow from haem *d* to haem *b*_595_. The use of extinction coefficients for the difference (reduced-*minus*-oxidized) spectra of haem *b*_595_ and haem *d* [62] makes it possible to gain an estimate of the extent of the fast electron transfer: within 0.2–1.5 μs 3–4% of haem *d* is oxidized and the same amount of haem *b*_595_ is reduced. Earlier, the electron backflow from haem *d* to haems *b* could only be observed on the slower, microsecond time scale with the one-electron-reduced *bd*-type oxidase from *Azotobacter vinelandii* [63] and *E*. *coli* [7] at low CO concentrations.

**Fig 4:**
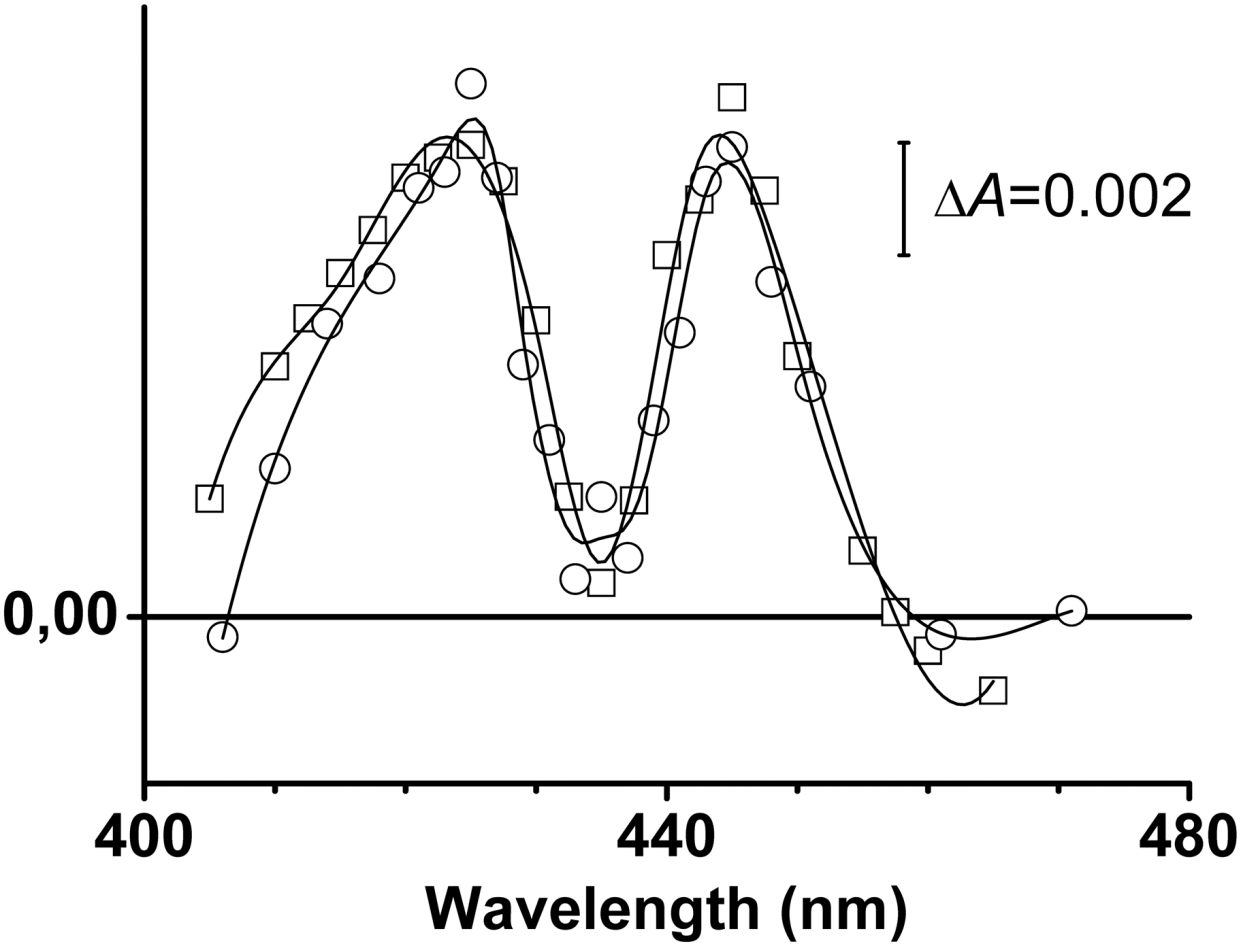
CO photolysis from haem *d* in the fully reduced cytochrome *bd*-I. Transient spectra at delay times of 200 ns (square symbols) and 1.5 μs (circle symbols).

Transient absorption spectra at delay times of 200 ns and 1.5 μs following CO photodissociation from the fully reduced cytochrome *bd*-I are shown by Fig 4 as a control. The spectra are very similar. They have a typical “W” (reversed) line shape with two maxima at ca. 425 and 445 nm and a minimum at about 435 nm. Such a peculiar “W” line shape is typical of static difference absorption spectra of CO binding to haem *d* in the fully reduced *bd*-type oxidase [32, 34, 64, 65]. Therefore, these photoinduced spectral changes (Fig 4) can be associated with the removal of CO from haem *d* triggered by flash photolysis of the haem *d* Fe-CO bond. This control shows that, in contrast to the one-electron-reduced cytochrome *bd*-I, photodissociation of CO from haem *d* in the fully reduced oxidase does *not* lead to any intraprotein electron transfer between the metal-containing redox cofactors. This is expected because, in the dithionite-reduced cytochrome *bd*-I, all the haem groups are in the ferrous oxidation state (Fe^2+^).

Thus, the new observation is the lack of the photoinduced electron transfer between haem *d* and haem *b*_595_ at 200 ns in one-electron-reduced cytochrome *bd*-I. Internal electron-transfer reactions seem to occur on a slower time scale.

It should be noted that the amplitude of the reverse electron transfer depends on the difference in apparent midpoint redox potentials (*E*_m_) of haems *d* and *b*_595_. The *E*_m_ values of haems *d* and *b*_595_ obtained by spectroelectrochemical equilibrium redox titration were reported to be +265 and 165 mV respectively [62]. Accordingly, their difference (Δ*E*_m_) is about 100 mV. According to the Nernst equation, such a large difference should correspond to a very small extent (~2%) of the electron backflow expected. The amplitude of the electron backflow observed in this work is in agreement with the estimated one. Taking the measured value (~3.5%) into account, the difference in the transient *E*_m_ values of the haems is calculated to be 84 mV.

In haem-copper oxidases, the total amplitude of the reverse electron transfer (including the 1.4-ns and 3-*μ*s phases, and the 35-*μ*s phase of the electron transfer from a low-spin haem and a binuclear center to Cu_A_) varies from ~4% (cytochrome *ba*_3_ from *Thermus thermophilus* [66]) to ~25–35%, ~50% and ~75%—in cytochromes *aa*_3_ from bovine heart [67], *Paracoccus denitrificans* [68] and *Rhodobacter sphaeroides* [47, 69], respectively. The amplitude of the electron backflow of the 1.4-ns phase itself is ~6% in cytochrome *bo*’ from *E*. *coli* [49] and 11–16% in bovine heart cytochrome *aa*_3_ [46], and as in case of cytochrome *bd*-I, is modulated by the difference in redox potentials of electron carriers. Thus, the observed extent of the electron backflow in cytochrome *bd*-I is not due to a damaged fraction of the preparation but is defined by Δ*E*_m_ of the two haems.

The rate constant for electron transfer from haem *d* to haem *b*_595_ should be the sum of the microscopic constants of forward (*k*_for_) and backword (*k*_back_) reactions. Assuming that the phase of electron transfer corresponds to the full equilibration between haem *d* and haem *b*_595_, ~3.5% amplitude of the reaction yields the equilibrium constant, K of ~28. Taking into account that the rate constant is the sum of the microscopic rate constants of forward (*b*_595_ → *d*) and backward (*d* → *b*_595_) electron transfer (*k*_for_ + *k*_back_) and that the equilibrium constant is *K = k*_for_/k_back_, the values for *k*_for_ and *k*_back_ can be estimated to be 0.48×10^6^–4.8×10^6^ s^-1^ and 0.17×10^7^–1.7×10^7^ s^-1^, respectively. Thus, the apparent value of the rate constant should be close to the *k*_for_.

For a long time, in the haem-copper terminal oxidases, the 3-*μ*s phase of the electron backflow was considered as the most rapid interhaem electron transfer process [48, 70–72]. Subsequently, it was proved that the 3-*μ*s event is preceded by a much faster 1.4-ns phase of the reverse electron transfer from haem *a*_3_ to haem *a* in bovine heart cytochrome *c* oxidase [46] or 1.2-ns phase of that from haem *o*' to haem *b* in the related quinol oxidase *bo*' of *E*. *coli* [49].

A phase of the rapid (0.2–1.5 μs) electron transfer between the haems in cytochrome *bd*-I reported here can be compared with these two consecutive kinetic components of the electron backflow reaction in the haem-copper oxidases. As shown earlier, the value of the rate constant of the 1.4-ns phase of the electron backflow in haem-copper oxidases is in fundamental agreement with the calculations for the activationless electron tunneling between the haems, based on the semiempirical Moser-Dutton rule [73, 74] and the known distance between the haem cofactors, the specific low value of the reorganization energy and the correctly calculated the average protein packing density [46, 49, 75, 76].

As noted before [77], when the donor—acceptor distance becomes short, as is the case for the haem—haem electron transfer in the haem—copper oxidases, the local heterogeneity of the coupling medium and the significantly reduced number of effective electron transfer pathways should be taken into account [49, 77, 78]. A more detailed model based on the quantum mechanical calculation of the coupling medium between donor and acceptor (the so-called pathway model) combined with the molecular dynamics enabled researchers to identify and describe theoretically the main pathways of the coupling between haem *a* and haem *a*_3_ of the bovine enzyme [79, 80].

With the present data on cytochrome *bd*-I, only very rough correlation between the rates of the fast interhaem electron transfer in the *bd* oxidase and the *aa*_3_ oxidase and the distances between the corresponding haems can be made. Apart from the unknown values of the reorganization energies, packing density and the coupling medium in cytochrome *bd*-I, there is an ambiguity in defining distance metrics between the haems (edge-to edge or Fe-to-Fe) caused by the fact that atoms on the periphery of the large porphyrin rings are not always well coupled to the central metal [76, 81, 82].

As pointed out earlier [81], because of the different efficiency of the coupling of the intervening polypeptide medium between the redox centers, the electron transfer rates at the same distance can differ by as much as a factor of 10^3^, whereas the donor/acceptor distances that differ by as much as 5 Å can yield identical rates. Since the fast reverse electron transfer reaction in cytochrome *bd*-I is about 2 orders slower than that in the haem-copper oxidases, the rate constant of the electron transfer between haem *d* and haem *b*_595_ is in agreement with the predicted Fe-Fe centre distance between the haems *d* and *b*_595_ (~10 Å) [36] that is close to the Fe-Fe centre distance between the haems *a* and *a*_3_ in haem-copper oxidases (~13 Å) [83].

An alternative possibility could be that the fast electron transfer between haem *d* and haem *b*_595_ in cytochrome *bd*-I is not limited by the pure electron tunneling between the haems, as in the case of the 3-*μ*s phase of electron backflow in the haem-copper oxidases. In contrast to the 1.4-ns phase, the 3-*μ*s electron transfer is the phase with a significant activation barrier [45]. Its rate is limited by the CO migration from Cu_B_ out of the protein that is accompanied by a slight modification of the *a/a*_3_ redox equilibrium [46]. It would be expected that since cytochrome *bd*-I is lacking Cu_B_, the dissociation of CO should not limit this component of the electron transfer. However, the absence of the copper ion does not exclude a possibility of a transient trapping of CO on its way from haem *d* to the outside which, if coupled to the structural rearrangement, may theoretically control change in redox potentials of the haems *d* and *b*_595_ and the electron transfer between them.

According to the modeling of excitonic interactions between ferrous haems *d* and *b*_595_ in the absorption and circular dichroism spectra [36] and the data from this work, the Fe-Fe distance between haems *d* and *b*_595_ should be much larger than that between haem *a*_3_ and Cu_B_ in cytochrome *c* oxidase (4.5 Å [84] or 5.2 Å [85]). If this is the case, we cannot consider haem *d* and haem *b*_595_ as forming a binuclear center in cytochrome *bd* for O_2_ reduction in which haem *b*_595_ is a functional analogue of Cu_B_. However, haem *b*_595_ could play a role to provide haem *d* with an electron [40] and possibly a proton in the course of the O_2_ reduction reaction.

**Fig 5:**
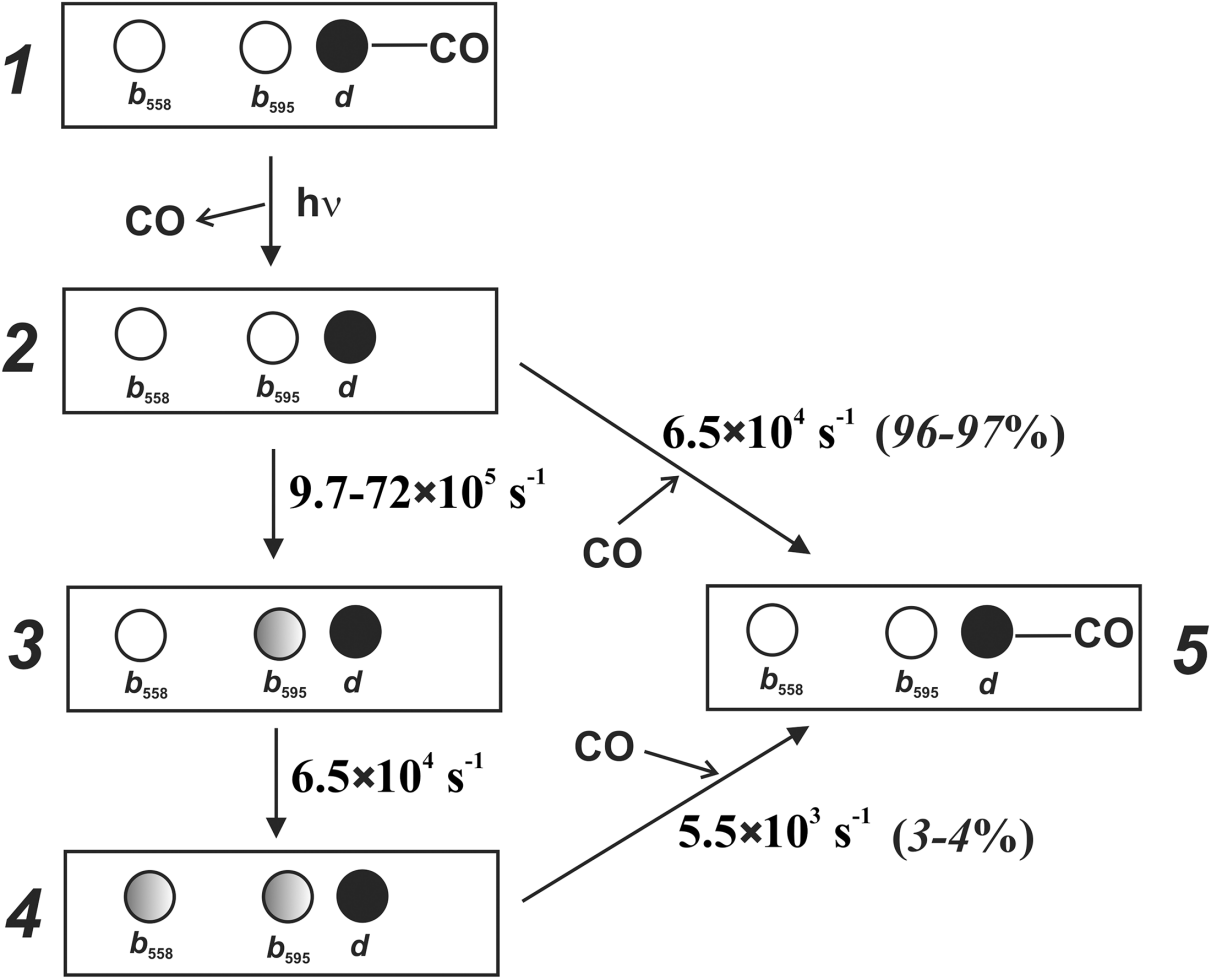
Reverse electron transfer within one-electron-reduced cytochrome *bd*-I following photodissociation of CO. This scheme is based on the current work and on the works of Rappaport et al. [37] and Siletsky et al. [39]. Circles denote haems. Open circle shows that haem is oxidized. Black filled circle shows that haem is reduced. Gray filled circle shows that a small fraction of haem (≤4%) is transiently reduced. Laser flash causes time-unresolved dissociation of CO from haem *d* (1→2 transition). At CO concentration of about 1 mM, in most of the hemoprotein molecules in which ultrafast geminate recombination of CO with chlorin has not occurred [33, 35, 37] CO returns to haem *d* with the apparent first-order rate constant (*k*) of 6.5×10^4^ s^-1^ at 1 mM CO (2→5 transition, right branch). However, in 3–4% of the enzyme population photodissociation of CO is followed by electron transfer from haem *d* to haem *b*_595_ with *k* in the range 9.7×10^5^ to 7.2×10^6^ s^-1^ (2→3 transition). Then the electron transfers to haem *b*_558_ with *k* of 6.5×10^4^ s^-1^ (3→4 transition). After that, the electron is probably redistributed between the haems in accordance with their midpoint redox potentials [62]. Eventually, in this small fraction of the hemoprotein the electron returns from haem *b*_558_ and haem *b*_595_ to haem *d* simultaneously with recombination of CO with chlorin with the apparent first-order rate constant (*k*) of 5.5×10^3^ s^-1^ (4→5 transition).

Fig 5 shows the proposed scheme of electron backflow reaction in cytochrome *bd*-I that is based on the present and previous reports [37, 39]. In brief, a laser flash induces dissociation of CO from the one-electron-reduced state of the enzyme that is followed by electron redistribution between the haem groups in 3–4% of the oxidase population. The electron from haem *d* first moves to haem *b*_595_ and then reaches haem *b*_558_. Subsequently, CO recombines back to haem *d* that is coupled to the return of the electron from haems *b*_558_ and *b*_595_ to haem *d*.

Our present results thus indicate that the electron transfer between the two catalytically relevant haem groups (haem *d* and haem *b*_595_) of cytochrome *bd*-I cannot itself limit or control catalysis of the oxygen reduction, and that the mechanism of nanosecond interhaem electron transfer may be universal not only in the family of haem—copper oxidases but also in the *bd*-type terminal oxidases. The placement of haem *b*_595_ close enough to the haem *d* catalytic site is to assure that electron transfer into the site will be rapid enough to provide the fast reduction of molecular oxygen without the release of potentially harmful partly-reduced oxygen intermediates. Besides, the rapid electron transfer may also help to control the potential reactivity of the haem *d* porphyrin radical that arises in the catalytic cycle upon formation of the ‘P’ state [9, 86] by quickly reducing it in the energy-coupled electron transfer.

## Conclusions

The reverse electron transfer from haem *d* to the haems *b* induced by flash-photolysis of one-electron-reduced CO-bound cytochrome *bd*-I of *E*. *coli* consists of two kinetically different phases. The well-defined electron equilibration between the haems with τ ~16 μs appeared to be preceded by the previously unnoticed fast electron tunneling from haem *d* to haem *b*_595_ in the time interval between 0.2 and 1.5 μs. The major novel finding of this study is that there is no electron transfer at 200 ns. These data are consistent with the suggestion that haem *b*_595_ is required for rapid electron donation during catalysis and provide insights into mechanisms of enzymatic O_2_ reduction coupled to energy conservation.
